# An anatomical and radiological study of the tectorial membrane and its clinical implications

**DOI:** 10.1038/s41598-022-25213-2

**Published:** 2022-12-12

**Authors:** Shin Hyo Lee, Tae-Hyeon Cho, Hyun-Jin Kwon, Ju Eun Hong, Young Han Lee, Hun-Mu Yang

**Affiliations:** 1 Translational Laboratory for Clinical Anatomy, Department of Anatomy, Yonsei University College of Medicine, 50 Yonsei-ro, Seodaemun-gu, Seoul, 03722 Republic of Korea; 2 Department of Biomedical Laboratory Science, College of Software and Digital Healthcare Convergence, Yonsei University MIRAE Campus, Wonju, Republic of Korea; 3 Department of Radiology, Center for Clinical Imaging Data Science, Research Institute of Radiological Science, Yonsei University College of Medicine, Seoul, 03722 Republic of Korea; 4 Surgical Anatomy Education Centre, Yonsei University College of Medicine, Seoul, Republic of Korea

**Keywords:** Anatomy, Medical research

## Abstract

The radiological image of an intact tectorial membrane (TM) became an important favorable prognostic factor for craniovertebral instability. This study visualized the fascial layers of the TM and adjacent connective tissues with clinical significance by micro-CT and histological analysis. The TM firmly attached to the bony surface of the clivus, traversed the atlantoaxial joint posteriorly, and was inserted to the body of the axis showing wide distribution on the craniovertebral junction. The supradental space between the clivus, dens of the axis, anterior atlantooccipital membrane, and the TM contained profound venous networks within the adipose tissues. At the body of the axis, the compact TM layer is gradually divided into multiple layers and the deeper TM layers reached the axis while the superficial layer continued to the posterior longitudinal ligament of the lower vertebrae. The consistent presence of the fat pad and venous plexus in the supradental space and firm stabilization of the TM on the craniovertebral junction was demonstrated by high-resolution radiologic images and histological analysis. The evaluation of the TM integrity is a promising diagnostic factor for traumatic craniovertebral dislocation.

## Introduction

The craniovertebral junction (CVJ) is a biomechanical and anatomic unit that includes the occiput and the upper two cervical vertebrae (atlas and dens of the axis)^1^. The ligaments of the CVJ inevitably play an important role in preserving the stability of the region, protecting the neural and vascular structures passing this junction while simultaneously allowing significant mobility^2,3^. Approximately one-third of all cervical spine injuries involve the CVJ^4^. Consequently thorough knowledge about the complex ligamentous anatomy of the CVJ is a prerequisite for accurate interpretation of the neural injuries in upper cervical trauma^5^.

The intrinsic ligaments which act as main stabilizers of the CVJ form three layers anterior to the dura mater including the odontoid ligaments (alar and apical ligaments), the cruciate ligament, and the tectorial membrane (TM)^6,7^. Among the main ligaments of the CVJ, the cruciate and the alar ligaments between the occiput and the median atlantoaxial joint have uniformly been described in the literature^8,9^. On the contrary, the detailed anatomy of the TM, another stabilizer of the CVJ remains inconsistent and the least explored of the intrinsic ligaments^10,11^.

The TM runs posterior to the cruciate ligament is intimately in contact with the dura mater, as a cranial continuation of the posterior longitudinal ligament supporting whole vertebrae^2,10^. In recent literature, intact TM appears to be an important favorable prognostic factor in atlantooccipital dislocation which is considered to be a frequently fatal injury^12^. Fiester et al. have proposed that the alteration of the supradental space (fat-filled space superior to the atlantoaxial joint, inferior to the basion of the clivus, and anterior to the TM) followed by injury of the TM may indicate a patient with significant CVJ instability^13–16^.

Osseous injury can be subtle while representing important radiological red flags for significant underlying ligamentous injury^17^. In the cases without the overt sign of subluxation or dislocation of the atlantooccipital joint, early diagnosis of unstable CVJ injuries by the ligamentous structures and treatment with post-surgical stabilization has shown to greatly reduce neurological deterioration and death^7^. Describing the various imaging appearances of the TM will increase the familiarity of TM tear patterns for the clinicians and help better predict the potential for unstable CVJ injuries and thus guide appropriate clinical management^18^. This potential of the TM in the clinical field and paucity of the basic research on the TM prompt us to investigate the structure of the TM by an alternative method for image analysis and histological observations. This study aimed to provide clear radiological images of the CVJ soft tissues by micro-CT and describe histological characteristics of the TM along with the cranium and upper cervical vertebrae that could contribute to the understanding of CVJ injuries.

## Methods

### Subjects

Five embalmed cadavers (3 men and 2 women) with a mean age of 76.6 ± 8.0 years were dissected in this study. The donors signed documents agreeing to their participation in the body donation program of the applicable medical school. The study obtained appropriate consent to use cadavers legally donated to the Surgical Anatomy Education Centre of the Yonsei University College of Medicine (approval No. 22-002). This study was conducted in accordance with the Declaration of Helsinki.

Specimens having visible signs of deformity and operative procedures in the CVJ were excluded. In the prone position, the anatomists removed the skin, fascia, and muscles overlying the CVJ. The occiput and the posterior lamina of the atlas were removed by saw. Laminectomies of the axis, C3, and C4 were then conducted to remove the brainstem and spinal cord and exposed the ligamentous complex of the CVJ. The continuum of the clivus–atlas-axis was preserved intact and separated from the retropharyngeal space. Three cadavers were subjected to 3D topographic analysis of the micro-CT images and histological revalidation by the longitudinal section. Specimens from one cadaver were subjected to histological evaluation by transverse section. One cadaver was used for the noncalcified plastic section of the whole CVJ and conventional histological observations.

### Micro-CT preparation and 3D visualization

Our micro-CT preparation technique for enhancing soft-tissue contrast was used. Briefly, the whole specimen of the CVJ including the clivus–atlas–axis-C3 from three embalmed cadavers were soaked in 1% phosphotungstic acid solution with 70% ethanol after serial dehydration (30%, 50%, and 70% for 24-h each). Micro-CT images were acquired using a micro-CT scanner (Skyscan 1173, Bruker, Kontich, Belgium), with a source voltage of 70 kVp, source current of 114 μA, and image pixel of 20 μm^2^. For a 3D visualization and analysis, the acquired section images were reconstructed using CTVox (Bruker, Kontich, Belgium). Representative 3D images of the TM are provided in online Supplemental Material 1.

### Histology

Bony structures with intact soft tissues were cropped from clivus, dens of the axis, and body of the axis from each specimen. The tissue blocks were decalcified and processed for routine paraffin embedding. Longitudinal and transverse sections of the tissue block cut in 4 µm thickness were subjected to H&E, Masson’s Trichrome, and Verhoeff Van Giseon staining. To produce non-decalcified longitudinal sections of the CVJ including the clivus–atlas–axis-C3, a specimen was stored in 70% ethanol, dehydrated in a series of 80%, 95%, and 100% ethanol baths, and then cleared with acetone. The specimen was infiltrated with methyl methacrylate and polymerized. Plasticized blocks were then micro-ground and polished to approximately 70 μm thick using an Exakt Micro Grinding System. Polished samples were stained with Goldner’s trichrome.

### Ethics statements

The study design was approved by the Institutional Review Board of Yonsei University Health System, though they exempted this study from a formal review due to the use of cadaver specimens. The study obtained appropriate consent to use cadavers legally donated to the Surgical Anatomy Education Centre of the Yonsei University College of Medicine.

## Results

**Figure 1:**
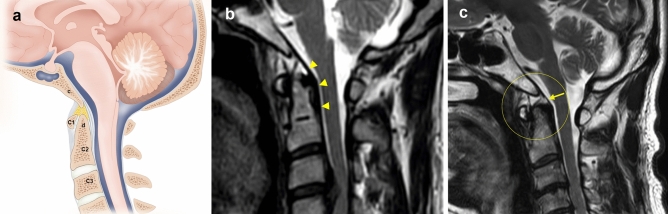
Sagittal view of the craniovertebral junction. (**a**) The cruciate ligament–tectorial membrane (TM)–dura mater complex between the clivus and the body of the axis. *c* clivus, *d* dens. (**b**) Sagittal T2-weighted magnetic resonance image shows thin normal delineated TM with normal integrity from clivus to dens (arrowheads). (**c**) Sagittal T2-weighted magnetic resonance image shows a discontinuous TM (asterisks) surrounded by fluid signal intensity. In a patient with TM tear, the TM shows attachment to the superior clivus. The TM tear is visualized at the level of apex of the dens (arrow). At the level of the body of the axis, the TM shows irregular fraying, and the continued posterior longitudinal ligament is retracted with coarse attachment to the dens.

An inclined part of the occipital bone anterior to the foramen magnum sustains the midbrain as the clivus. The TM traversed the inner surface of the clivus and upper cervical vertebrae, surfacing to the brainstem and spinal cord, and continued to the posterior longitudinal ligament in the inferior body of the axis, or intervertebral space between axis and C3 (Fig. 1a).

**Figure 2:**
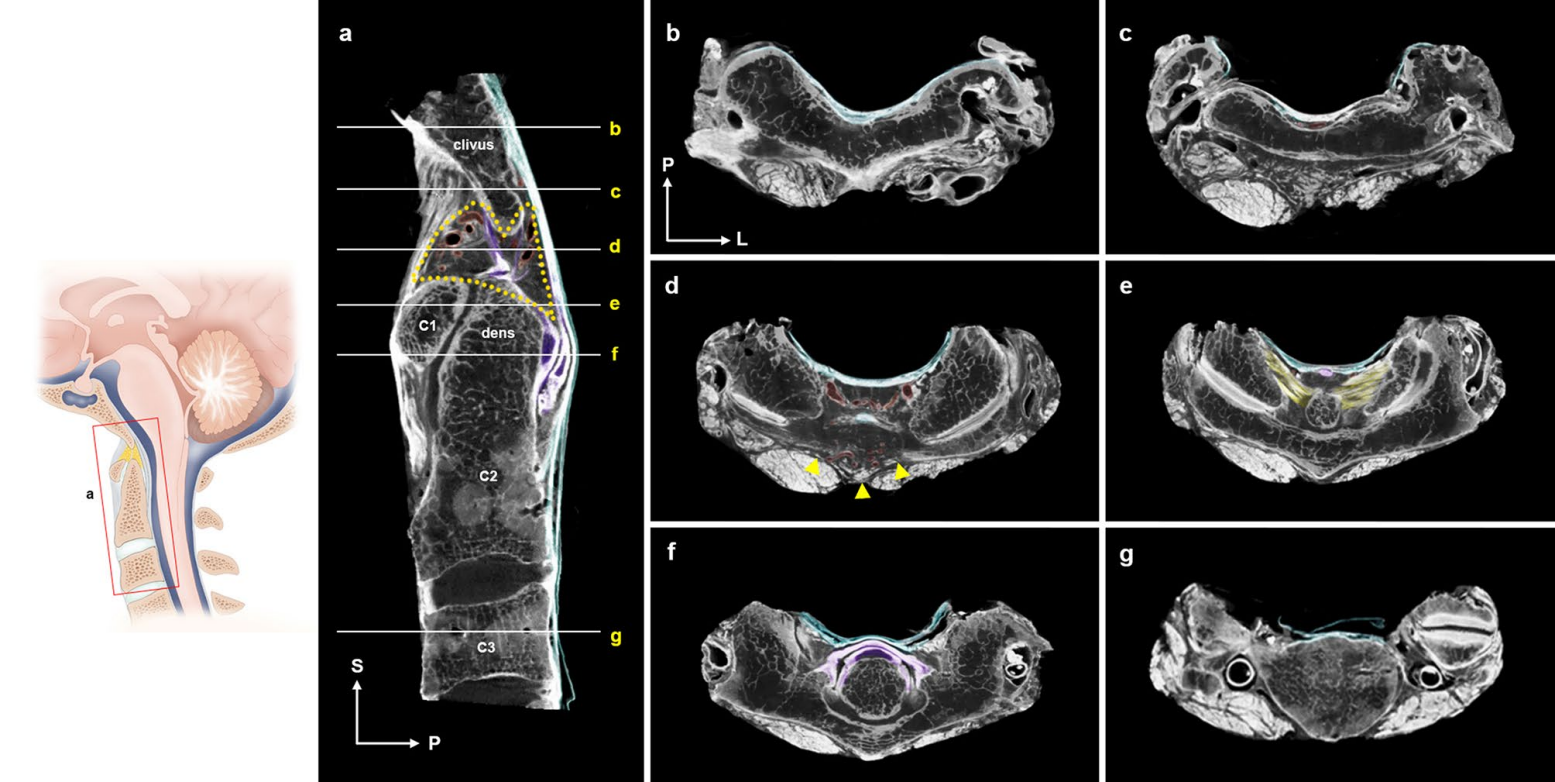
Micro-CT images of the craniovertebral junction (CVJ). (**a**) Mid-sagittal view of the CVJ. The supradental space (yellow dotted line) with dilated venous plexus (red area). (**b**) Coronal view of the firmly attached tectorial membrane (TM) to the periosteum of the superior clivus (blue area). (**c**) Coronal view of inferior clivus. Vessels (red area) from the infra-clival region invaded the niche formed by the TM departing from the inferior clivus. (**d**) The supradental space between clivus and dens. Note profound venous plexus (red area) in hypointense adipose tissues posterior to the anterior atlantooccipital membrane (arrowheads). (**e**) Ligamentous structures at the level of apex of the dens are attached by an alar ligament (yellow area). The superior crus of the cruciate ligament (purple area) is anterior to the TM. (**f**) The transverse ligament of cruciate ligament posterior to the hypertrophied cartilage of dens (purple area) and medial osculation between the TM and dura mater (blue area). (**g**) Medial attachment of the posterior longitudinal ligament to the body of C3 and separated spinal dura mater (blue area).

The neural image by micro-CT provides clear identification of the soft tissues of the CVJ compared to the clinical magnetic resonance image (Figs. 1b,c, and 2).

Ligamentous structures of the CVJ in this study observed by sagittal view could be described as follows: the anterior atlantooccipital membrane (AAOM) in the foremost part, the apical ligaments between the plateau of dens and the clivus, and the cruciate ligament–TM–dura mater complex in the posterior part (Fig. 2a). The TM is firmly attached to the bony surface of the clivus showing wide distribution to the jugular tubercle (Fig. 2b). The venous plexus in the loose connective tissues gradually invaded the space between the inferior clivus and the cruciate ligament–TM–dura mater complex (Fig. 2c). The supradental space between the clivus, dens of the axis, AAOM, and TM contained profound venous networks within the loose connective tissues (Fig. 2a,d). The alar ligament bilaterally connected the superior dense to the occipital condyle served as the lateral boundary of the supradental space (Fig. 2e). The cruciate ligament has been distinguished from the TM below the impingement site of the dens to the cruciate ligament–TM–dura mater complex (Fig. 2f). From the level of the C3, the TM was continued as the posterior longitudinal ligament (Fig. 2g).

**Figure 3:**
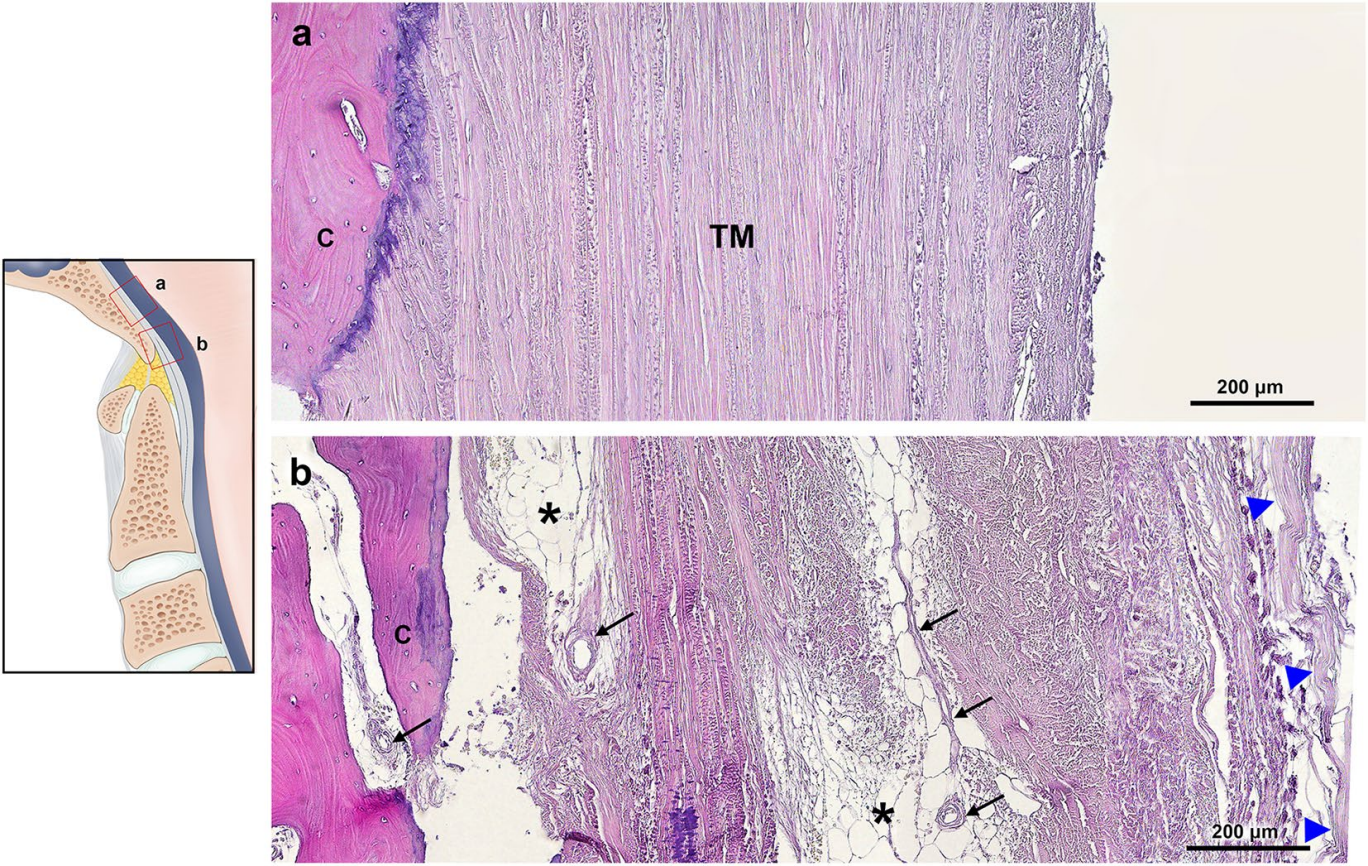
Attachment of tectorial membrane (TM) in the clivus. H&E stain. (**a**) The superior clivus by longitudinal section. Aligned fibers of the TM are firmly interdigitating to the bone, indistinguishable from the superior crus of the cruciate ligament and the cranial dura mater. (**b**) Multiple layers of ligamentous structures in the inferior clivus demarcated by adipose tissues (asterisks) and vessels (arrows). *Arrowheads* dura mater layer, *c* clivus, *TM* tectorial membrane.

In the superior clivus, collagen fibers of the superior crus of cruciate ligament–TM–dura complex firmly attached to the bony surface in an interdigitated manner (Fig. 3a). On the inferior clivus, this complex became distinguishably intermingled with adipose tissues and vessels. The collagen bundles exhibited some directional change with abundant fiber branching (Fig. 3b).

**Figure 4:**
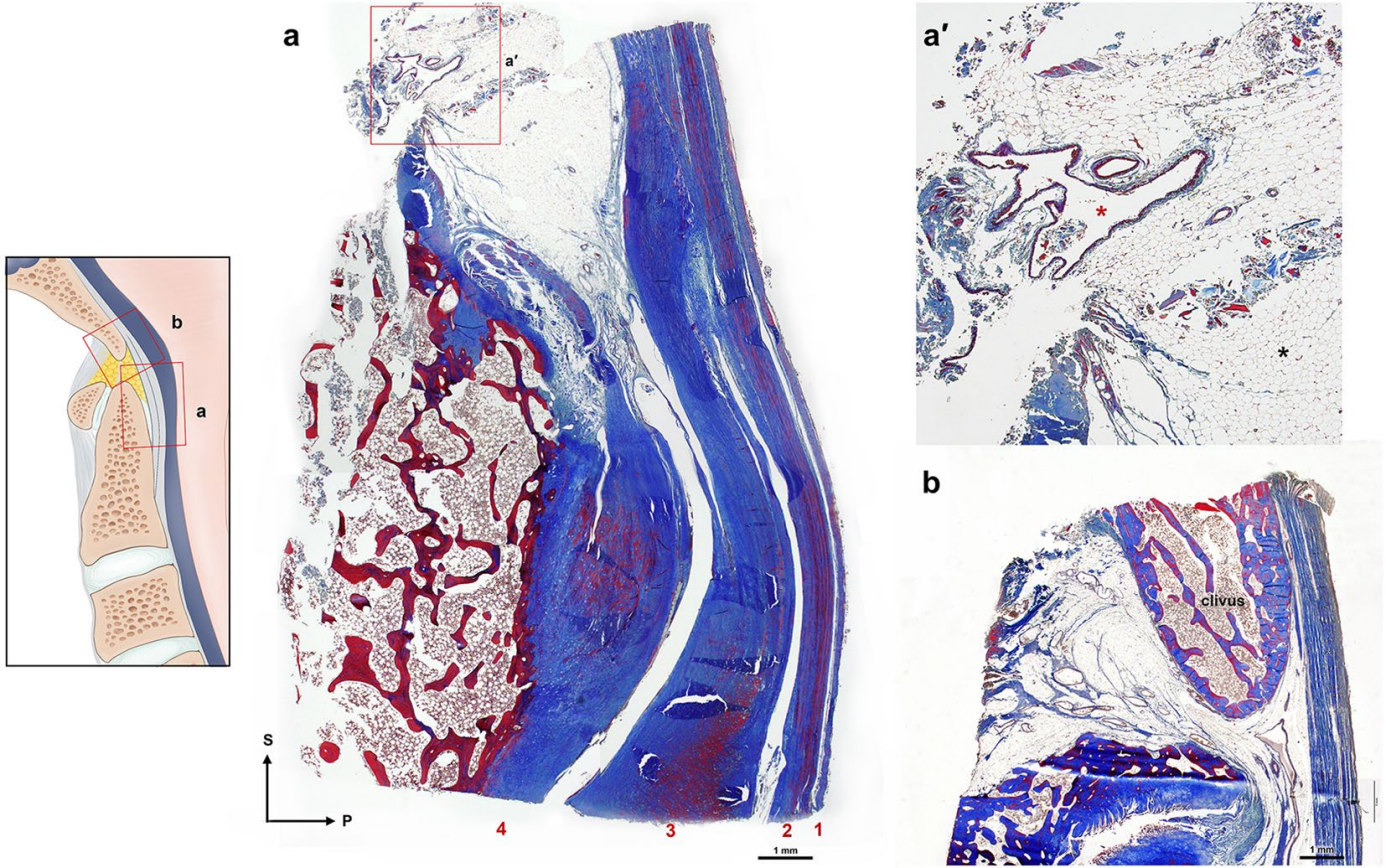
The tectorial membrane (TM) in the sub-clival level. Masson’s trichrome stain. (**a**) Sagittal view of the sub-clival area of the cruciate ligament–TM–dura mater complex. 1, spinal dura mater; 2, TM; 3, cruciate ligament; 4, hypertrophied cartilage of the dens. (**a**′) Magnification of a box in (**a**). The venous plexus (red asterisk) in the adipose tissues (black asterisk) of the supradental space. (**b**) The cruciate ligament–TM–dura mater complex departs from the inferior clivus and forms the posterior boundary of the supradental space filled with adipose tissues and venous plexus intermingled with sparse fibrous tissues (arrows).

As described above, since the impingement site to dens of the axis the cruciate ligament–TM–dura mater complex was distinguishable by each layer by micro-CT analysis. Between the hypertrophied cartilage on the plateau of dens and the superior crus of the cruciate ligament, the fat pad was always present. This supradental space contains extensive venous plexuses intermingled with multiple fibrous septa expanded to the bony surface of the other ligamentous structures (Fig. 4).

**Figure 5:**
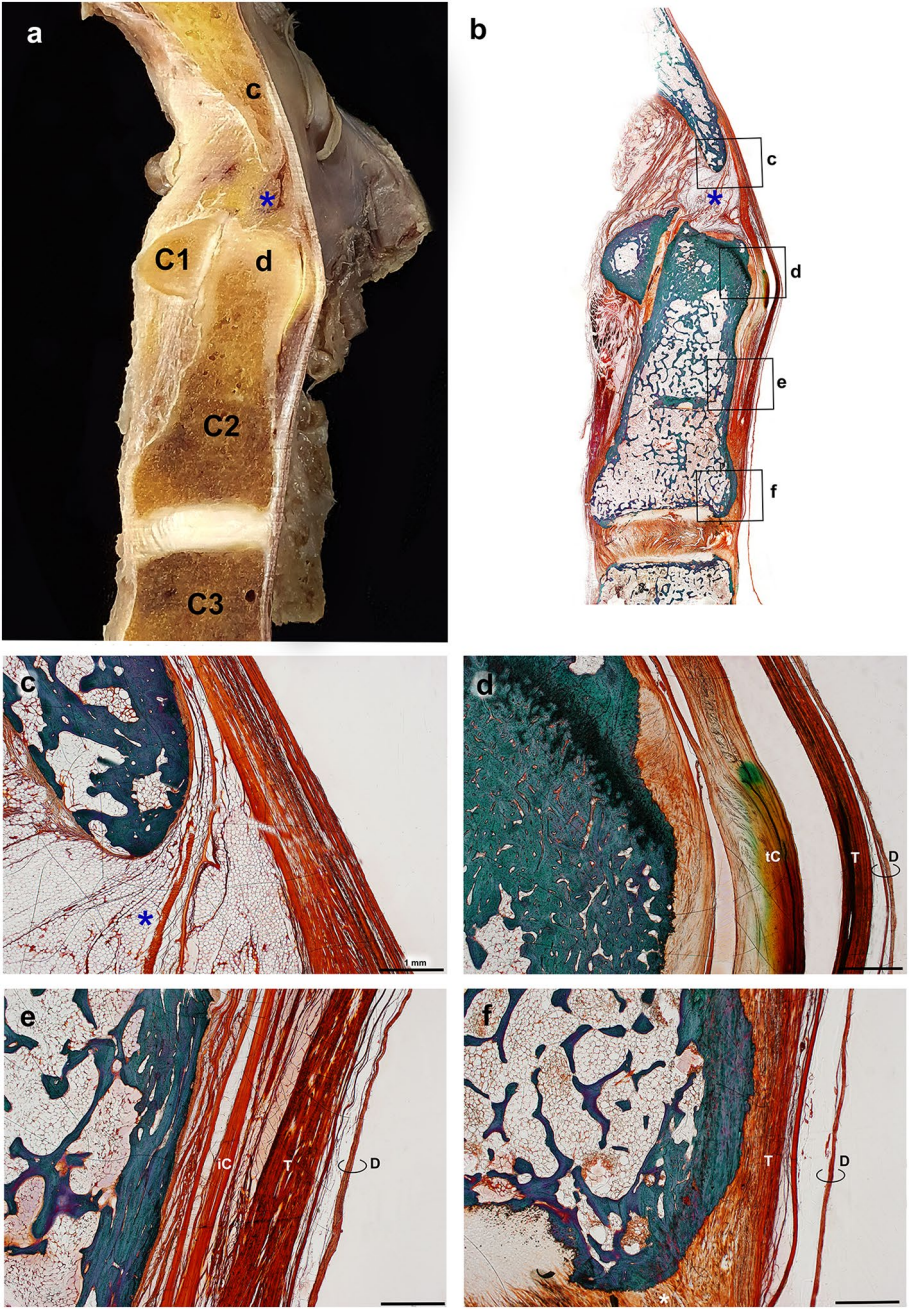
Sagittal view of the craniovertebral junction. Gross hemisection (**a**) and a plastic embedded section by Goldner’s Trichrome stain (**b**) of the same specimen. Dark orange stain indicates dense connective tissues attached to the bony tissue (green stain). *c* clivus, *d* dens. (**c**–**f**) Show magnifications of boxes of a whole specimen. (**c**) The inferior clivus forms the superior border of the supradental space (asterisk) filled with adipose tissues. (**d**) The cruciate ligament–tectorial membrane (TM)–dura mater complex in the impingement site of a dens. Arrows indicate a calcified transverse ligament of the cruciate ligament. (**e**) The fibers of the inferior crus of the cruciate ligament are close to the TM in the body of the axis. (**f**) A deep layer of the TM is firmly attached to the connective tissues of the intervertebral space between the axis and C3 (white asterisk), except for the superficial layer of the TM and spinal dura mater. *D* dura mater, *iC* inferior crus of the cruciate ligament, *tC* transverse ligament of the cruciate ligament, *T* tectorial membrane.

The morphology of the TM abruptly changed by region, as cornified tissues in the post-dental space, loosely arranged multiple layers in the insertion to the body of the axis and densely packed deep layer of the TM attached to the connected tissues of the intervertebral space between the axis and C3 (Fig. 5).

**Figure 6:**
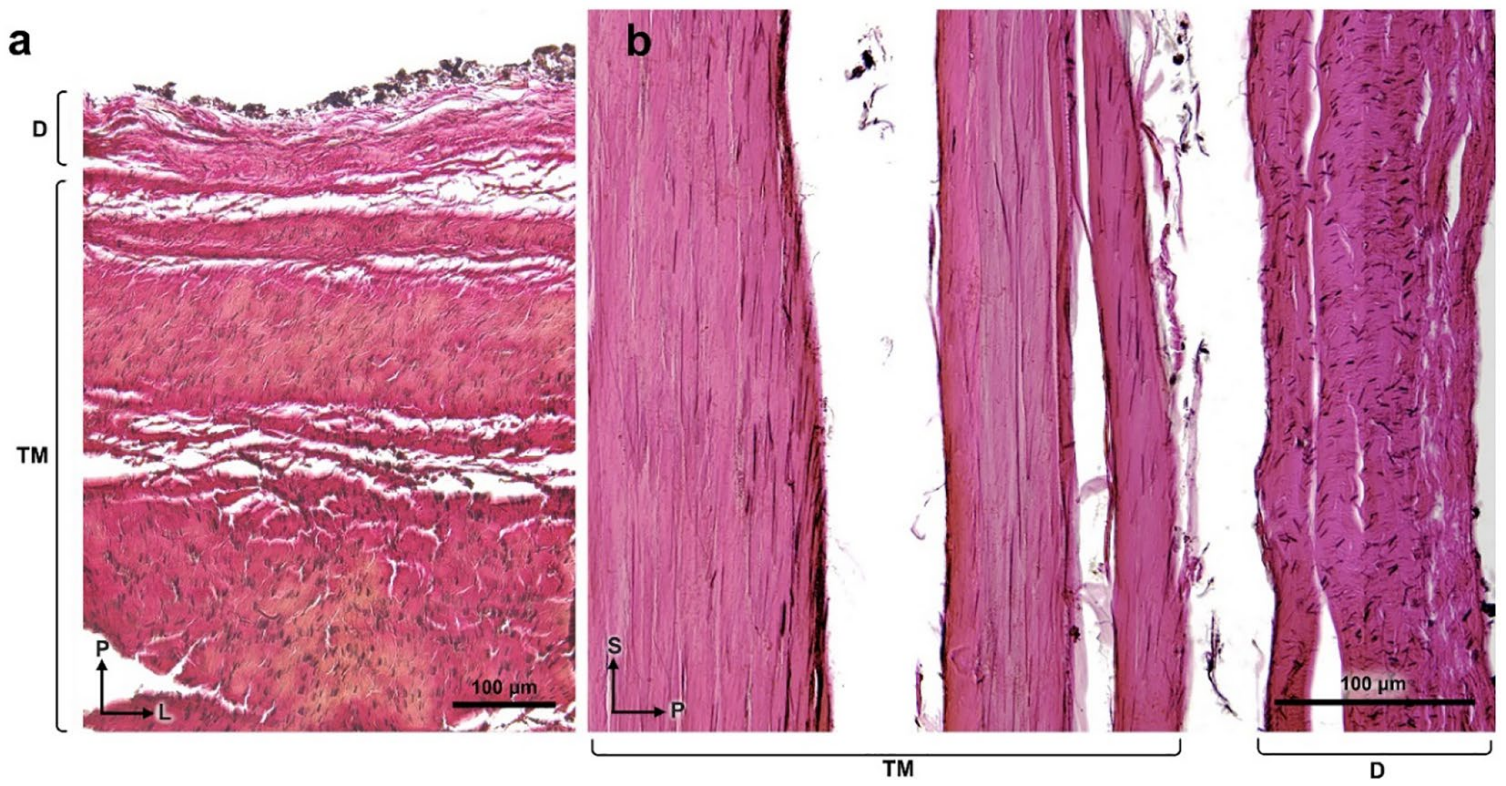
Distribution of elastic fibers in the tectorial membrane (TM) and the dura mater. Verhoeff Van Giseon stain. (**a**) A cross-section of ligamentous tissues is firmly attached to the superior clivus. Longitudinal thick elastic fibers (black spots) are identified in the deep layer of the TM, contrary to the cranial dura mater (D). (**b**) A longitudinal section of ligamentous tissues at the level of the sub-clival region. The spinal dura mater contains horizontal elastic fibers. The TM showed longitudinal elastic fibers (black lines) parallel to the collagen bundles (pink). *D* dura mater, *TM* tectorial membrane.

The TM contains dense regular connective tissues with an abundance of collagen fibers and interspersed elastic fibers. In the cranial origin of the TM, elastic fibers ran parallel to the longitudinal collagen bundles. In the TM layers, a deep layer of the TM adjacent to the bony surface of the clivus contained longitudinal thick elastic fibers (Fig. 6a). In the cervical vertebral level, the middle layer of the TM showed a marginal increase and central decrease of elastic fibers parallel to the collagen bundles. The spinal dura mater consists of multi-biased networks of numerous elastic fibers compared to the sparse elastic fibers of the cranial dura mater (Fig. 6b).

## Discussion

Visualization of the cervical ligaments by micro-CT allows the contrast to the bone and the soft tissues including the vessels and loose connective tissues to be better observed. This study has materialized the high-resolution image of cadaveric human CVJ using micro-CT and validated the association between these radiological and histological features. As an early prognostic factor of neural injury, intact features of the fascial layers of the TM and anterior ‘supradental space’ were also depicted by histological observation.

To date, outcome prognostication in patients with an atlantooccipital dislocation has been based mainly on clinical indicators and osseous relationships on radiographs or cervical CT scans^18^. Recently, the TM regarded as a secondary stabilizer of the atlantooccipital junction has been a critical determinant as to whether a CVJ injury is stable or unstable since the evaluation of tectorial integrity allows for supplementary prognostication, and may facilitate the decision to opt for early surgical intervention^12^. The epidural space between the TM and the clivus (supradental space) is predominantly fat-filled recess superior to the atlanto-axial joint and inferior to the basion of the clivus^14^. This is a potential space that contains a small venous plexus and may become filled with blood if the clivus is fractured or the TM is injured^17^. In the setting of significant cervical hyperflexion-hyperextension injury in adult patients, the TM may tear with a resultant shearing injury of the adjacent venous plexus in the supradental space causing the “supradental sign”—the hematoma formation and effacement of the fat pad in the supradental space^19^. The supradental sign by imaging analysis is proved by histologic observations of the cruciate ligament–TM–dura mater complex departs from the inferior clivus. The ligamentous structure including the TM forms the posterior boundary of the supradental space filled with adipose tissues and profound venous plexus which is visible both by micro-CT and histologic sections. The adipose tissues compartmented by thin fibrous septa were widely distributed between the occiput and dens as a fat pad and posteriorly supported by the TM. The typical presence of the fat pad in the supradental space directly visualized on CT and MRI exams allows improvement of the sensitivity for diagnosing major CVJ injuries and further expedite follow-up cervical MRI to evaluate the remaining major CVJ ligaments for injury^20^. Although the venous plexus in this space has been thought to communicate with the extradural venous plexus and drained into the intraspinal plexus^21–24^, studies on their exact origins are insufficient. Further insights into the venous network of the supradental space could help the surgeons avoid troublesome venous bleeding during the transoral surgical approaches for the CVJ intervention^24^.

According to in vitro experimental study, the TM could be irreversibly stretched, especially when the head is forced to both flex and axially rotate, despite the atlantoaxial joint being the most mobile portion of the spine^25,26^. To confirm this property on the morphologic base, this study investigated the directions of fibers and elastic distributions on the TM. From the cranial origin to the insertion to the body of the axis, one-directional elastic fibers of the TM ran parallel to the longitudinal collagen bundles. At the sub-clival level, interspersed elastic fibers between collagen bundles decreased compared to that of the attachment site to clivus. Contrary to the TM layers, the spinal dura mater contained an increased number of multi-biased networks of elastic fibers compared to the sparse elastic fibers of the cranial dura mater. During the rotation of the CVJ, the dura mater encompassing the spinal cord may stretch multiple directions while the TM firmly stabilizes the atlantoaxial joint by restricting excessive rotation. Meanwhile, the TM at the level of the atlantooccipital joint and atlantoaxial joint was relatively condensed and stiff. As it reaches the body of the axis, the compact TM layer is gradually divided into multiple layers and the deeper TM layers are inserted to the axis in concurrence with the coarse attachment of the inferior crus of the cruciate ligament. Relatively incomplete supports of the ligamentous structures including the TM to the axis could explain an improved outcome of posterior C1–C2 fusion with Goel–Harms technique in the case of atlantoaxial dislocation^27^. Superficial TM layers become narrowed and elongated as the posterior longitudinal ligament on the body of C3. In other words, the ligamentous structure of the CVJ abruptly decreases at the level of the intervertebral disc and the body of C3 as the two main stabilizers (the cruciate ligament and TM) end in the axis. This result may explain the reason those surgical interventions at the C2–C3 level is associated with a higher rate of approach-related morbidity^28^.

## Conclusion

This study visualized the fascial layers of the TM and the supradental space with clinical significance by both micro-CT and histological analysis. The evaluation of the TM integrity is a promising diagnostic factor for traumatic CVJ instability without a distinct sign of CVJ dislocation.

## Supplementary Information

Supplementary Video 1.

Supplementary Information.

Supplementary Legends.

## Data Availability

The datasets used and/or analysed during the current study available from the corresponding author on reasonable request.
