# Author Correction: An anatomical and radiological study of the tectorial membrane and its clinical implications

**DOI:** 10.1038/s41598-023-28998-y

**Published:** 2023-01-31

**Authors:** Shin Hyo Lee, Tae‑Hyeon Cho, Hyun‑Jin Kwon, Ju Eun Hong, Young Han Lee, Hun‑Mu Yang

**Affiliations:** 1 Translational Laboratory for Clinical Anatomy, Department of Anatomy, Yonsei University College of Medicine, 50 Yonsei‑ro, Seodaemun‑gu, Seoul, 03722 Republic of Korea; 2 Department of Biomedical Laboratory Science, College of Software and Digital Healthcare Convergence, Yonsei University MIRAE Campus, Wonju, Republic of Korea; 3 Department of Radiology, Center for Clinical Imaging Data Science, Research Institute of Radiological Science, Yonsei University College of Medicine, Seoul, 03722 Republic of Korea; 4 Surgical Anatomy Education Centre, Yonsei University College of Medicine, Seoul, Republic of Korea

Correction to: *Scientific Reports* 10.1038/s41598-022-25213-2, published online 12 December 2022

The Funding section in the original version of this Article was incomplete.

“This work was supported by the National Research Foundation of Korea (NRF) grant funded by the Korean government (No. 2020R1F1A1058123). No other external funding or competing interests were declared.”

now reads:

“This work was supported by the National Research Foundation of Korea (NRF) grant funded by the Korean government (No. 2020R1F1A1058123) and a faculty research grant of Yonsei University Medicine for 6-2021-0043. No other external funding or competing interests were declared.”

The original Article has been corrected.

