# Supplementary material for: An anatomical and radiological study of the tectorial membrane and its clinical implications
Source: Sci Rep. 2022 Dec 12;12:21480. doi: 10.1038/s41598-022-25213-2 (PMC9744818; doi:10.1038/s41598-022-25213-2)
Supplement: Supplementary file 2 — Supplementary Information. [file 41598_2022_25213_MOESM2_ESM.docx]

|  |  |
| --- | --- |
|  | Micro-CT images of the craniovertebral junction |
|  | Coronal view of ligamentous structures including the tectorial membrane, cruciate ligament, and alar ligament |
|  |  |
