# Supplementary material for: An anatomical and radiological study of the tectorial membrane and its clinical implications
Source: Sci Rep. 2022 Dec 12;12:21480. doi: 10.1038/s41598-022-25213-2 (PMC9744818; doi:10.1038/s41598-022-25213-2)
Supplement: Supplementary file 3 — Supplementary Legends. [file 41598_2022_25213_MOESM3_ESM.docx]

**Supplementary Material 1.**

3D sectional image by micro-CT correspondent to **Figure 2b–g**. D, dens; L, lateral; OC, occipital condyle; P, posterior.
